# Pb^2+^-Containing Metal-Organic Rotaxane Frameworks (MORFs)

**DOI:** 10.3390/molecules26144241

**Published:** 2021-07-13

**Authors:** Ting Xia, Zhi-Yong Yu, Han-Yuan Gong

**Affiliations:** 1 Department of Chemistry, Renmin University of China, No. 59, Zhongguan Street, Beijing 100872, China; ruc_xia.ting-2018102281@ruc.edu.cn; 2 College of Chemistry, Beijing Normal University, No. 19, Xinwai Street, Beijing 100875, China

**Keywords:** metal-organic rotaxane frameworks, MORF, Texas-sized molecular box, Pb^2+^

## Abstract

The metal-organic rotaxane framework (MORF) structures with the advantage of mechanically interlocking molecules (MIMs) have attracted intense interest from the chemical community. In this study, a set of MORFs (i.e., MORF-Pb-1 and MORF-Pb-2) are constructed using Pb^2+^, a tetraimidazolium macrocycle (Texas-sized molecular box; **1^4+^**), and aromatic dicarboxylate (*p*-phthalate dianions (PTADAs; **2**) or 2,6-naphthalene dicarboxylate dianions (**3**)) via a one-pot three-layer diffusion protocol. In particular, an unusual Pb…Pb weak interaction was shown in MORF-Pb-1 (charactered with distance of 3.656 Å).

## 1. Introduction

**Figure 1:**
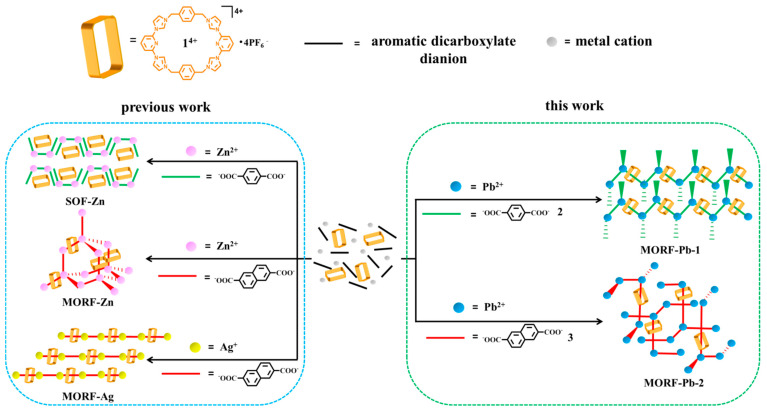
Schematic representation of metal-organic rotaxane framework (MORF) and supramolecular organic framework (SOF) formed with metal cation (Pb^2+^, Zn^2+^, or Ag^+^) in the presence of **1^4+^**, *p*-phthalate dianions (PTADAs, **2**), or 2,6-naphthalene dicarboxylate dianions (**3**).

Metal-organic framework (MOF) materials have potential applications in a variety of fields, such as gas storage [1,2,3,4], heterogeneous catalysis [5], sensors [6], luminescence material [7], etc. Specifically, metal-organic rotaxane framework (MORF) structures involve mechanically interlocking molecules (MIMs; i.e., rotaxanes) [8,9,10,11,12] and attract interest of the chemical community. To date, crown ether [13,14,15,16,17,18], “blue box” (CBPQT^4+^) [19,20], cucurbiurils [21,22,23], octametallic metallacrown [24,25,26,27], and polyamide [28,29] have been utilized to produce a series of MORF structures. The Texas-sized molecular box ([cyclo[2](2,6-di(1*H*-imidazol-1-yl)pyridine)[2](1,4-dimethylenebenzene), **1^4+^**) [30,31] was developed to construct MORFs cooperating with metal cations (e.g., Zn^2+^, Ag^+^, or lanthanide) and aromatic dicarboxylate anions (Figure 1).

Recently, Pb-containing materials have received widespread attention, including perovskite materials and novel lead batteries. The reported Pb-containing MOFs present different applications in gas storage [32,33,34], catalysis [35,36,37], electrode materials [38], high-energy density materials [39,40], luminescence materials [34,37,41], DNA probes [42], etc. Surprisingly, to the best of our knowledge, it is still a challenge to effectively construct MORF materials with Pb metal cation participation. Herein, we demonstrated that Pb^2+^ can cooperate with the Texas-sized molecular box (**1^4+^**) and aromatic dicarboxylate anions (e.g., *p*-phthalate dianions (PTADAs; **2**) or 2,6-naphthalene dicarboxylate dianions (**3**)) to construct the first class of Pb^2+^-containing MORF structures.

## 2. Results and Discussion

A facile multicomponent self-assembly-based approach was developed to generate Pb^2+^-containing MORFs. It is a one-pot three-layer diffusion protocol. As detailed below, Pb^2+^ cation (Pb(NO_3_)_2_; 0.120 mL of 0.05 M solution in H_2_O) was added as the first layer of three separate layers within a separate vial. A mixture of DMF and H_2_O (3 mL; 1:1, *v*/*v*) was then added as the middle layer. A premixed solution (containing **1^4+^**•4PF_6_^−^ (0.0036 M), *p*-phthalic acid (2H^+^•**2**; 0.0143 M), and NMe_4_^+^•OH^−^•5H_2_O (0.0286 M) in a mixture of DMF and H_2_O (0.84 mL; 1:1, *v*/*v*)) was then added as the upper layer. Standing for two weeks, [**1^4+^**•(**2**)_4_•(2H^+^•**2**)•Pb_2_•2DMF•12H_2_O] (namely MORF-Pb-1) was obtained as single crystals suitable for X-ray diffraction analysis. When 2H^+^•**2** was replaced by 2,6-naphthalene dicarboxylic acid (2H^+^•**3**) in the same protocol, crystalline samples of the [**1^4+^**•(**3**)_5_•Pb_3_•12.5H_2_O] (MORF-Pb-2) were achieved. Single crystal analysis provided direct evidence for the formation of both Pb^2+^-containing MORF structures (Table 1).

### 2.1. Crystal Structure of [1^4+^•(2)_4_•(2H^+^•2)•Pb_2_•2DMF•12H_2_O] (MORF-Pb-1)

In MORF-Pb-1, a chain-shaped 1D polyrotaxane structure [**1^4+^**•(**2**)_4_•Pb_2_]_n_ was constructed via the complexation between Pb(1) and **2** (highlighted in magenta colour). The 1D polyrotaxane further form a 2D and 3D array stabilized by intermolecular π-π donor–acceptor interactions and a possible unusual Pb…Pb weak interaction.

**Figure 2:**
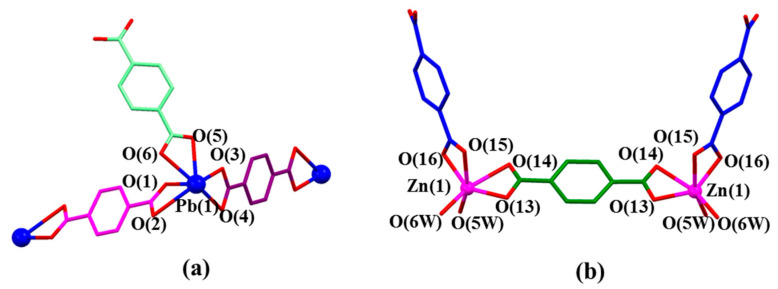
(**a**) Coordination mode of Pb(1) metal and surrounding O donors in the single crystal structure of [**1^4+^**•(**2**)_4_•(2H^+^•**2**)•Pb_2_•2DMF•12H_2_O] (MORF-Pb-1). Selected interatomic distances [Å] for Pb(1) complexation: Pb(1)…O(1) 2.33(4), Pb(1)…O(2) 2.82(5), Pb(1)…O(3) 2.36(4), Pb(1)…O(4) 2.87(1), Pb(1)…O(5) 2.46(3), Pb(1)…O(6) 2.61(6); Selected interatomic angles: O(1)…Pb(1)…O(2) 49.8(5)°, O(1)…Pb(1)…O(3) 81.8(1)°, O(1)…Pb(1)…O(4) 77.2(0)°, O(1)…Pb(1)…O(5) 83.0(0)°, O(1)…Pb(1)…O(6) 77.0(3)°, O(2)…Pb(1)…O(3) 124.5(1)°, O(2)…Pb(1)…O(4) 89.8(6)°, O(2)…Pb(1)…O(5) 120.7(8)°, O(2)…Pb(1)…O(6) 81.1(9)°, O(3)…Pb(1)…O(4) 48.9(5)°, O(3)…Pb(1)…O(5) 67.7(6)°, O(3)…Pb(1)…O(6) 116.9(2)°, O(4)…Pb(1)…O(5) 115.3(6)°, O(4)…Pb(1)…O(6) 152.3(2)°, O(5)…Pb(1)…O(6) 51.2(2)°; (**b**) Coordination mode of Zn(1) metal and surrounding O donors in [(**1^4+^**)_2_•(**2**)_9_•Zn_6_•12H_2_O]•2OH^-^•88.5H_2_O [44]. Note: Different colours are used for the linkage anions to illustrate the different local chemical environments.

In the structure of MORF-Pb-1, Pb^2+^ cations locate in the same environment. As Figure 2a shows, Pb(1) coordinates with three *p*-phthalate dianions (**2**) in different local chemical environments. Pb^2+^ has an outmost electronic closed shell as 5d^10^6p^2^ and a reported ionic radius of 1.33 Å. [43] It was compared with the other closed outmost shell Zn^2+^ cation (3d^10^, ionic radius of 0.74 Å) in the presence of **1^4+^** and **2**. Swapping Pb^2+^ with Zn^2+^, the same procedure resulted in [(**1^4+^**)_2_•(**2**)_9_•Zn_6_•12H_2_O]•2OH^−^•88.5H_2_O [44]. These structures showed that each Zn^2+^ only binds with two *p*-phthalate dianions (**2**) and cannot cooperate with **2** inserting into the cavity of **1^4+^** to form MIMs. All these findings show that the interactions between Pb^2+^ and **2** and the further MORF-Pb-1 construction are highly metal cation dependant.

**Figure 3:**
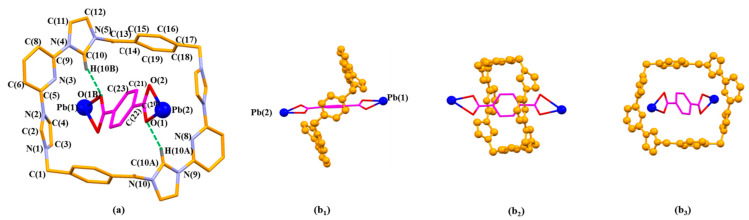
The pseudorotaxane unit [**1^4+^**•**2**•Pb_2_]^6+^ in the single crystal X-ray structure of [**1^4+^**•(**2**)_4_•(2H^+^•**2**)•Pb_2_•2DMF•12H_2_O] (MORF-Pb-1) shown in the stick form with atom-labelling scheme (**a**). Top (**b_1_**), side (**b_2_**), and front (**b_3_**) views of the [**1^4+^**•**2**•Pb_2_]^6+^ pseudorotaxane structure are shown. Selected interatomic distances [Å] for possible C-H…π interactions: C(21)…C(15) 3.79(0), C(21)…C(16) 3.75(8), C(23)…C(14) 3.63(0), C(23)…C(15) 3.64(6); Possible intermolecular hydrogen bonding interactions are evidenced by the following selected interatomic distances [Å]: C(10A)…O(1) 3.10(2), C(10)…O(1B) 3.10(2) and selected interatomic angles: C(10A)-H(10A)…O(1) 148.0(2)°, C(10)-H(10B)…O(1B) 148.0(2)°.

One molecule of **2** threads through an individual **1^4+^** via C-H…π interactions between the bridge bezene planes on **1^4+^** and the aromatic ring of **2** highlighted in magenta in Figure 3a. The interpenetrated structure is further stabilized via intermolecular hydrogen bonding interactions (e.g., C(10A)-H(10A)…O(1) and C(10)-H(10B)…O(1B)) between **1^4+^** and **2**.

**Figure 4:**
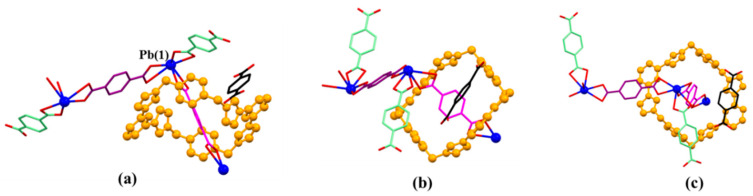
Top (**a**), side (**b**), and front (**c**) views of the monomer unit of MORF-Pb-1 formed from **1^4+^**, **2**, 2H^+^•**2**, and Pb^2+^. Note: Different colours are used for the linkage anions to illustrate the different local chemical environments. Solvent molecules and the protons have been omitted for clarity; none of these components are involved in the framework structure.

The asymmetric monomer unit of MORF-Pb-1 contains one molecule of macrocycle **1^4+^**, three Pb^2+^, three PTADA ligands in different local chemical environments, and two coordinated H_2_O solvent moieties. In particular, one molecule of free *p*-phthalic acid molecule is located in the channels of MORF-Pb-1 (Figure 4).

**Figure 5:**
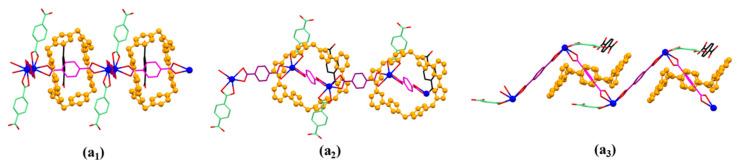
Top (**a_1_**), front (**a_2_**), and side (**a_3_**) views of the 1D polyrotaxane structure unit of MORF-Pb-1 found in the single crystal structure of [**1^4+^**•(**2**)_4_•(2H^+^•**2**)•Pb_2_•2DMF•12H_2_O] (MORF-Pb-1).

Monomer units can further construct a chain-shaped 1D polyrotaxane structure that extends infinitely in the direction of the crystallographic *a*-axis through the complexation between Pb(1) and **2** (highlighted with dark magenta colour) (Figure 5).

**Figure 6:**
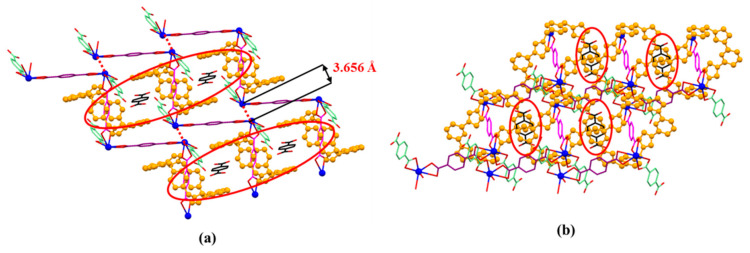
The front (**a**) and side (**b**) views of 2D-array shown in MORF-Pb-1. Terephthalic acid (2H^+^•**2**) molecule (highlighted with black colour) inserted into the cavity of MORF-Pb-1 via strong π-π donor–acceptor interactions.

It is found that the distance between two neighbouring Pb^2+^ on different 1D polyrotaxane is 3.656 Å (shown in Figure 6a), which is larger than the sum of two Pb^2+^ ionic radii (1.33 Å). To the best of our knowledge, the maximum distance between two Pb metals in the lead cluster is 3.07 Å. [45,46] Herein, the weak Pb…Pb interaction is suggested to be similar to halogen bonds and to further stabilize the 2D array formed with polyrotaxanes in [**1^4+^**•(**2**)_4_•(2H^+^•**2**)•Pb_2_•2DMF•12H_2_O]. To the best of our knowledge, it is the first example of a halogen bond-like interaction shown in the Pb metal cation form. The characterization of these Pb…Pb interactions is under further investigation.

**Figure 7:**
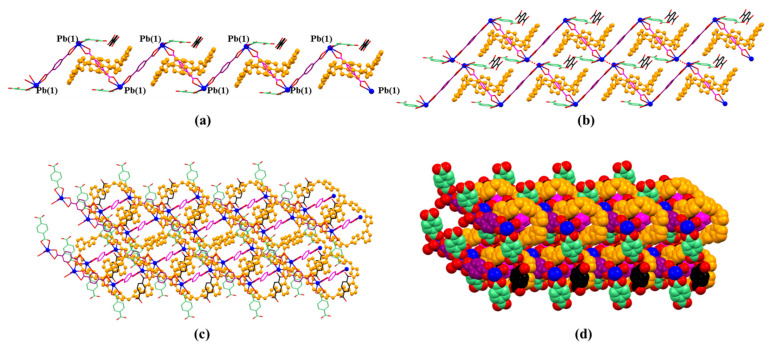
(**a**) 1D polyrotaxane, (**b**) 2D polyrotaxane network structure present within the single crystal X-ray structure of [**1^4+^**•(**2**)_4_•(2H^+^•**2**)•Pb_2_•2DMF•12H_2_O] (MORF-Pb-1). Overall structure as wire frame (**c**) or space filling (**d**) form representations of the polyrotaxane 3D array within the single crystal X-ray structure of [**1^4+^**•(**2**)_4_•(2H^+^•**2**)•Pb_2_•2DMF•12H_2_O] (MORF-Pb-1). Note: Different colours are used for the linkage anions to illustrate the different local chemical environments.

It is noted that free *p*-phthalic acid (2H^+^•**2**) molecules are located in the channels of MORF-Pb-1. The distances between bezene on 2H^+^•**2** and the planes on two neighbouring **1^4+^** are less than 3.6 Å. The finding implies that the π-π donor–acceptor interactions further stabilized the 2D array. Furthermore, the 2D layers shown above are organized via strong π-π donor–acceptor interactions between neighbouring **1^4+^** on different 2D layers. Finally, the 3D array of MORF-Pb-1 was achieved (Figure 7).

### 2.2. Crystal Structure of [1^4+^•(3)_5_•Pb_3_•12.5H_2_O] (MORF-Pb-2)

In MORF-Pb-2, a chain-shaped 1D polyrotaxane structure [**1^4+^**•(**3**)_5_•Pb_6_•H_2_O]_n_ was constructed via the complexation between Pb(4) and **3** (highlighted in green colour). The 1D polyrotaxane further forms 2D and 3D frameworks bridged with the coordination between Pb(1) and **3** (highlighted in black colour) and Pb(3, 5) and **3** (highlighted in black and purple colour), respectively.

**Figure 8:**
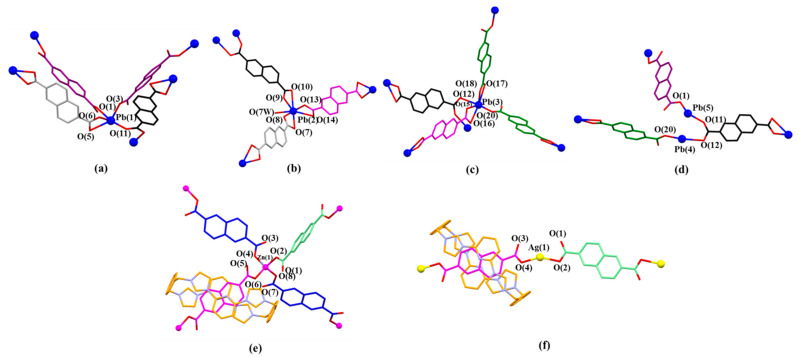
(**a**) Coordination modes of Pb(1) and surrounding O donors. Selected interatomic distances [Å] for metal cation Pb(1) complexation: Pb(1) …O(1) 2.37(7), Pb(1)…O(3) 2.39(7), Pb(1)…O(5) 2.78(1), Pb(1)…O(6) 2.39(5), Pb(1)…O(11) 2.57(9); Selected interatomic angles for these contacts are: O(1)…Pb(1)…O(3) 82.9(8)°, O(1)…Pb(1)…O(11) 154.02°, O(1)…Pb(1)…O(5) 73.74°, O(1)…Pb(1)…O(6) 73.81°, O(3)…Pb(1)…O(11) 76.5(2)°, O(3)…Pb(1)…O(6) 77.7(3)°, O(3)…Pb(1)…O(5) 127.4(9)°, O(5)…Pb(1)…O(11) 106.55°, O(5)…Pb(1)…O(6) 50.95°, O(6)…Pb(1)…O(11) 86.25°; (**b**) Coordination mode of Pb(2) metal and surrounding O donors. Selected interatomic distances [Å] for Pb(2) complexation: Pb(2)…O(10) 2.89(5), Pb(2)…O(9) 2.36(1), Pb(2)…O(13) 2.36(8), Pb(2)…O(14) 2.86(3), Pb(2)…O(7) 2.76(1), Pb(2)…O(8) 2.37(2), Pb(2)…O(7W) 2.89(8); Selected interatomic angles for these contacts are: O(7)…Pb(2)…O(8) 49.9(8)°, O(7)…Pb(2)…O(7W) 105.4(3)°, O(7)…Pb(2)…O(9) 119.1(6)°, O(7)…Pb(2)…O(10) 163.6(8)°, O(7)…Pb(2)…O(13) 84.0(5)°, O(7)…Pb(2)…O(14) 69.1(7)°, O(8)…Pb(2)…O(7W) 88.2(2)°, O(8)…Pb(2)…O(9) 70.1(3)°, O(8)…Pb(2)…O(10) 118.2(0)°, O(8)…Pb(2)…O(13) 84.9(2)°, O(8)…Pb(2)…O(14) 106.8(1)°, O(9)…Pb(2)…O(10) 48.1(1)°, O(9)…Pb(2)…O(13) 80.7(9)°, O(9)…Pb(2)…O(14) 129.0(1)°, O(9)…Pb(2)…O(7W) 79.5(5)°, O(10)…Pb(2)…O(13) 83.5(3)°, O(10)…Pb(2)…O(14) 109.5(7)°, O(10)…Pb(2)…O(7W) 83.5(4)°, O(13)…Pb(2)…O(14) 48.7(9)°, O(13)…Pb(2)…O(7W) 160.3(5)°, O(14)…Pb(2)…O(7W) 150.6(0)°; (**c**) Coordination mode of Pb(3) metal and surrounding O donors. Selected interatomic distances [Å] for Pb(3) complexation: Pb(3)…O(12) 2.79(6), Pb(3)…O(15) 2.37(4), Pb(3)…O(16) 2.74(8), Pb(3)…O(17) 2.84(6), Pb(3)…O(18) 2.34(5), Pb(3)…O(20) 2.46(3); Selected interatomic angles for these contacts are: O(12)…Pb(3)…O(15) 78.4(0)°, O(12)…Pb(3)…O(16) 94.3(2)°, O(12)…Pb(3)…O(17) 84.1(5)°, O(12)…Pb(3)…O(18) 93.7(4)°, O(12)…Pb(3)…O(20) 163.6(1)°, O(15)…Pb(3)…O(16) 49.3(4)°, O(15)…Pb(3)…O(17) 117.2(5)°, O(15)…Pb(3)…O(18) 74.0(1)°, O(15)…Pb(3)…O(20) 85.3(5)°, O(16)…Pb(3)…O(17) 166.3(2)°, O(16)…Pb(3)…O(18) 119.3(0)°, O(16)…Pb(3)…O(20) 76.3(9)°, O(17)…Pb(3)…O(18) 47.4(8)°, O(17)…Pb(3)…O(20) 101.6(5)°, O(18)…Pb(3)…O(20) 79.5(9)°. (**d**) Coordination mode of Pb(4) and Pb(5) metal and surrounding O donors. Selected interatomic distances [Å] for Pb(4) and Pb(5) complexation: Pb(4)…O(20) 2.46(3), Pb(4)…O(12) 2.79(6), Pb(5)…O(1) 2.37(7), Pb(5)…O(11) 2.57(9); Selected interatomic angles for these contacts are: O(20)…Pb(4)…O(12) 163.6(1)°, O(1)…Pb(5)…O(11) 154.0(2)°. (**e**) Coordination mode of Zn(1) metal and surrounding O donors in [**1^4+^**•(**3**)_4_•Zn_2_•6H_2_O] [47] for comparison. (**f**) Coordination mode of Ag(1) metal and surrounding O donors in [**1^4+^**•(**3**)_3_•Ag_2_•16H_2_O] [48] for comparison. Note: Different colours are used for the linkage anions to illustrate the different local chemical environments.

In the structure of MORF-Pb-2, Pb^2+^ has five different coordination modes, labelled as Pb(1, 2, 3, 4, or 5), different from [**1^4+^**•(**3**)_4_•Zn_2_•6H_2_O] (MORF-Zn) containing **1^4+^**, **3**, and Zn^2+^, while Zn^2+^ coordinates with three 2,6-naphthalene dicarboxylate dianions (**3**) in different local chemical environments [47]. Meanwhile, Ag^+^ (4d^10^, ionic radius as 1.26 Å) can cooperate with **1^4+^** and **3** to form [**1^4+^**•(**3**)_3_•Ag_2_•16H_2_O] [48]. In MORF-Ag, Ag^+^ coordinates with two 2,6-naphthalene dicarboxylate dianions (**3**) in different local chemical environments in [**1^4+^**•(**3**)_3_•Ag_2_•16H_2_O] [48] (Figure 8).

**Figure 9:**
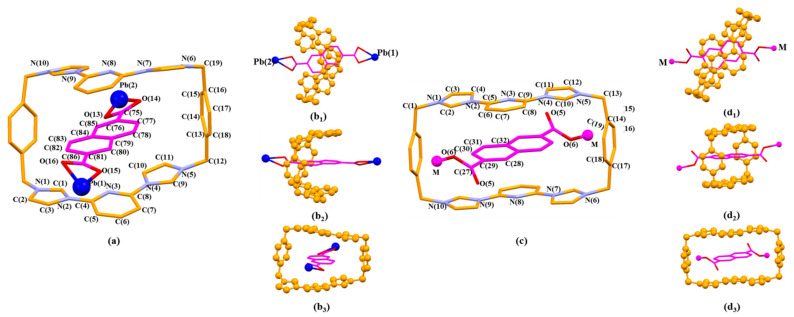
The pseudorotaxane unit [**1^4+^**•**3**•Pb_2_]^6+^ found in the single crystal X-ray structure of [**1^4+^**•(**3**)_5_•Pb_3_•12.5H_2_O] (MORF-Pb-2). Shown in the stick form with atom-labelling scheme (**a**). Top (**b_1_**), side (**b_2_**), and front (**b_3_**) views of [**1^4+^**•**3**•Pb_2_]^6+^ are shown here. Selected interatomic distances [Å] for possible π-π donor acceptor interactions: C(75)…C(11) 3.74(0), C(75)…C(10) 3.61(2), C(76)…C(10) 3.40(7), C(77)…C(10) 3.37(8), C(77)…C(11) 3.59(0), C(77)…N(4) 3.51(3), C(78)…C(8) 3.66(3), C(78)…N(4) 3.53(8), C(79)…N(3) 3.49(2), C(79)…C(8) 3.63(1), C(81)…C(5) 3.76(6), C(82)…C(3) 3.60(1), C(82)…N(2) 3.60(9), C(82)…C(4) 3.72(9), C(83)…C(3) 3.66(6), C(83)…N(2) 3.29(7), C(83)…C(4) 3.54(6), C(83)…C(1) 3.53(5), C(83)…N(3) 3.78(0), C(84)…N(3) 3.37(2), C(84)…C(4) 3.59(4), C(84)…N(2) 3.73(7), C(85)…N(3) 3.68(6). The pseudorotaxane unit [**1^4+^**•**3**•M_2_]^n+^ (M=Zn^2+^ or Ag^+^) for comparison (**b_1_**), (**b_2_**), (**b_3_**) found in the single crystal X-ray structure of [**1^4+^**•(**3**)_4_•Zn_2_•6H_2_O] (MORF-Zn) or [**1^4+^**•(**3**)_3_•Ag_2_•16H_2_O] (MORF-Ag) shown in the stick form with atom-labelling scheme (**c**). Top (**d_1_**), side (**d_2_**), and front (**d_3_**) views of [**1^4+^**•**3**•M_2_]^n+^ are shown here.

The pseudorotaxane structure in [**1^4+^**•**3**•Pb_2_]^6+^ (MORF-Pb-2) is also formed via π-π donor–acceptor interactions between **3** and **1^4+^**. However, the structure of MORF-Pb-2**, 1^4+^** is distorted and twisted into a “boat” configuration, while **1^4+^** adopts a more regular “box”-like configuration in MORF-Zn or MORF-Ag (Figure 9c; M = Zn^2+^ or Ag^+^).

**Figure 10:**
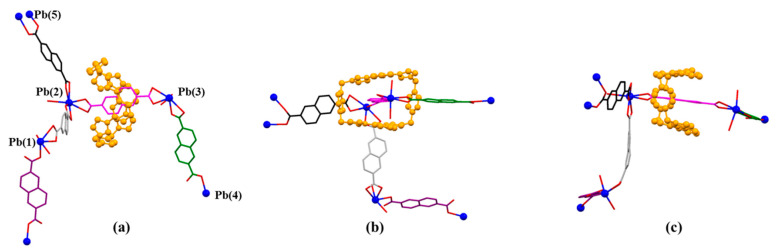
Top (**a**), side (**b**), and front (**c**) views of the monomer unit of MORF-Pb-2 formed from **1^4+^**, **3**, and Pb^2+^. Note: Different colours are used for the linkage anions to illustrate the different local chemical environments. Solvent molecules and the protons have been omitted for clarity; none of these components are involved in the framework structure.

The monomer unit of MORF-Pb-2 was found to include one molecule of macrocycle **1^4+^**, five Pb^2+^, five dicarboxylate ligands **3** in different local chemical environments, and one coordinated H_2_O solvent moiety (Figure 10).

**Figure 11:**
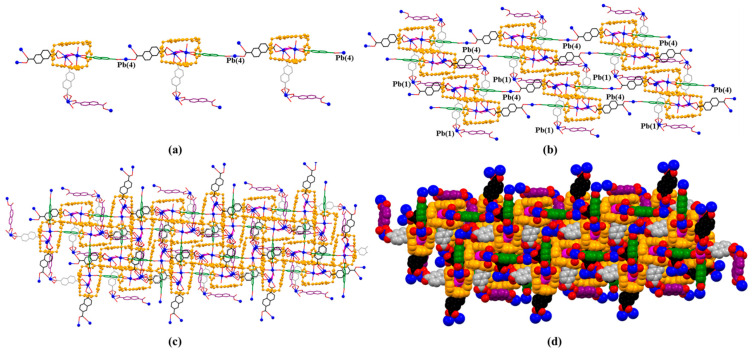
(**a**) 1D polyrotaxane, (**b**) 2D polyrotaxane network structure present within the single crystal X-ray structure of [**1^4+^**•(**3**)_5_•Pb_3_•12.5H_2_O] (MORF-Pb-2). Overall structure as wire frame (**c**), space filling (**d**) representations of the polyrotaxane 3D array. Note: Different colours are used for the linkage anions to illustrate the different local chemical environments.

Monomer units can construct a chain-shaped 1D polyrotaxane structure through the complexation between Pb(4) and **3** (highlighted in green colour, Figure 10a). The 1D polyrotaxane further forms 2D frameworks bridged with the coordination between Pb(1) and **3** (highlighted in black colour). Furthermore, the 2D layers shown above are organized to form the final 3D array with the coordination between Pb(3,5) and **3** (highlighted in black and purple colour) (Figure 11).

## 3. Materials and Methods

### 3.1. Reagents and Analytical Methods

For this study, all reagents were purchased commercially (Aldrich, Acros, or Fisher) and used without further purification. The single crystals used to obtain the X-ray diffraction structure grew as colourless plates with the .cif document available as a separate supporting information file providing details regarding the specific crystal used for the analysis, along with the structure in question. Diffraction grade crystals were obtained by slow evaporation from solution using a mixture of H_2_O/DMF as described below.

The data crystals were cut from a cluster of crystals and had the approximate dimensions given in the .cif document. The data were collected on a Saturn724+ (2 × 2 bin mode) or Mercury2 (2 × 2 bin mode) CCD diffractometer using a graphite monochromator with MoKα radiation. The structures were solved and refined by full-matrix least-squares on F^2^ with anisotropic displacement parameters for the non-H atoms using SHELXL-2014. [49] The hydrogen atoms were calculated in ideal positions with isotropic displacement parameters set to 1.2 × Ueq of the attached atom (1.5 × Ueq for methyl hydrogen atoms). The function, w (|Fo|^2^ − |Fc|^2^)^2^, was minimized. Definitions used for calculating R(F), Rw(F2), and the goodness of fit, S, are given below and in the .cif documents [49]. Neutral atom scattering factors and values used to calculate the linear absorption coefficient are from the International Tables for X-ray Crystallography (1992) [50]. All ellipsoid figures were generated using SHELXTL/PC [51]. Tables of positional and thermal parameters, bond lengths and angles, torsion angles, and figures and lists of observed and calculated structure factors are located in the .cif documents available from the Cambridge Crystallographic Data Centre (CCDC) via quoting ref. numbers 2084601 and 2084604. The document also contains details of crystal data, data collection, and structure refinement.

### 3.2. General One-Pot Three-Layer Diffusion Protocol

In a separate vial, Pb(NO_3_)_2_ (0.120 mL of 0.05 M solution in H_2_O) was added to form the first layer of three separate layers. A mixture of DMF and H_2_O (3 mL; 1/1, *v*/*v*) was added as the middle layer. A premixed mixture containing **1^4+^**•4PF_6_^−^ (0.120 mL of a 0.025 M solution in DMF), 2H^+^•**2** or 2H^+^•**3** (0.240 mL of a 0.05 M solution in H_2_O), and tetramethylammonium hydroxide pentahydrate (NMe_4_^+^•OH^−^•5H_2_O) (0.480 mL of a 0.05 M solution in H_2_O) was added as the upper layer. The final three-layer systems were set on the bench for two weeks, colourless crystals were cultivated from the clear solution, and these crystals proved suitable for an X-ray diffraction analysis.

## 4. Conclusions

In summary, Pb^2+^-containing MORF structures (i.e., [**1^4+^**•(**2**)_4_•(2H^+^•**2**)•Pb_2_•2DMF•12H_2_O] (MORF-Pb-1) or [**1^4+^**•(**3**)_5_•Pb_3_•12.5H_2_O] (MORF-Pb-2)) were constructed using Pb^2+^, a tetraimidazolium macrocycle (**1^4+^**), and *p*-phthalate dianions (**2**) or 2,6-naphthalene dicarboxylate dianions (**3**) in a one-pot three-layer diffusion protocol, separately. Compared with smaller Zn^2+^ and Ag^+^ with the outmost electronic closed shell, Pb^2+^ has more diverse coordination modes and benefits for metal-organic polyrotaxane formation. In particular, an unusual Pb…Pb weak interaction (characterised with distance of 3.656 Å) was shown in MORF-Pb-1. Further construction, property, and usability studies of Pb^2+^-containing MORFs are currently being evaluated.

## Figures and Tables

**Table 1:** X-ray crystallographic data summary of MORF-Pb-1 [**1^4+^**•(**2**)_4_•(2H^+^•**2**)•Pb_2_•2DMF•12H_2_O] and MORF-Pb-2 [**1^4+^**•(**3**)_5_•Pb_3_•12.5H_2_O].

	[1^4+^•(2)_4_•(2H^+^•2)•Pb_2_•2DMF•12H_2_O]	[1^4+^•(3)_5_•Pb_3_•12.5H_2_O]
**CCDC No.**	2084601	2084604
**Description**	prism	prism
**Colour**	colourless	colourless
**From solution**	DMF/H_2_O	DMF/H_2_O
**Empirical formula**	C_84_H_94_N_12_O_34_Pb_2_	C_98_H_89_N_10_O_32.5_Pb_3_
***Mr***	2230.09	2548.36
**Crystal size (mm^3^)**	0.11 × 0.07 × 0.04	0.21 × 0.12 × 0.10
**Crystal system**	triclinic	monoclinic
**Space group**	P -1	P 21/n
**a [Å]**	10.848(2)	20.504(4)
**b [Å]**	14.117(3)	20.005(4)
**c [Å]**	14.758(3)	24.046(5)
α **[deg]**	100.95(3)	90
β **[deg]**	101.97(3)	101.23(3)
γ **[deg]**	90.31(3)	90
**V/ [Å^3^]**	2168.3(8)	9674(3)
**d/[g/cm^3^]**	1.711	1.75
**Z**	1	4
***T*** **[K]**	173.15	153.15
**R1, wR2 I > 2ó(I)**	0.0462, 0.1398	0.0789, 0.1877
**R1, wR2 (all data)**	0.0485, 0.1426	0.1587, 0.2551
**Quality of fit**	1.008	1.006

## Data Availability

Crystallographic information files for MORF-Pb-1and MORF-Pb-2 can be obtained free of charge from the Cambridge Crystallographic Data Centre via www.ccdc.cam.ac.uk/data_request/cif (accessed on 8 June 2021) using the accession identifiers CCDC-2084601 and CCDC-2084604, respectively.

## Supplementary Materials

Electronic supporting information containing crystallographic tables and physicochemical characterisation data for MORF-Pb-1 and MORF-Pb-2 and crystallographic information files (CIFs) are available online.

## Sample Availability

Samples of the compounds MORF-Pb-1 and MORF-Pb-2 are available from the authors.
