# Supplementary material for: Pb2+-Containing Metal-Organic Rotaxane Frameworks (MORFs)
Source: Molecules. 2021 Jul 13;26(14):4241. doi: 10.3390/molecules26144241 (PMC8306753; doi:10.3390/molecules26144241)

checkCIF/PLATON report

Structure factors have been supplied for datablock(s) I

THIS REPORT IS FOR GUIDANCE ONLY. IF USED AS PART OF A REVIEW PROCEDURE FOR PUBLICATION, IT SHOULD NOT REPLACE THE EXPERTISE OF AN EXPERIENCED CRYSTALLOGRAPHIC REFEREE.

No syntax errors found. CIF dictionary Interpreting this report

Datablock: I

Bond precision: C-C = 0.0077 A Wavelength=0.71073

Cell:	a=10.848(2)	b=14.117(3)	c=14.758(3)
	alpha=100.95(3)	beta=101.97(3)	gamma=90.31(3)
Temperature:	173 K		

	Calculated	Reported
Volume	2168.3(8)	2168.3(8)
Space group	P -1	P -1
Hall group	-P 1	-P 1
Moiety formula	C16 H8 O8 Pb, 0.5(C38 H34 N10), 0.5(C8 H6 O4), C3 H7 N O, 6(O)	?
Sum formula	C42 H35 N6 O17 Pb	C84 H94 N12 O34 Pb2
Mr	1102.96	2230.09
Dx,g cm-3	1.689	1.708
Z	2	1
Mu (mm-1)	3.973	3.974
F000	1094.0	1118.0
F000’	1087.86	
h,k,lmax	14,18,19	14,18,19
Nref	9943	9929
Tmin,Tmax	0.723,0.853	0.000,0.000
Tmin’	0.639	

Correction method= # Reported T Limits: Tmin=0.000 Tmax=0.000
AbsCorr = NONE

Data completeness= 0.999 Theta(max)= 27.492

R(reflections)= 0.0462(9504) wR2(reflections)= 0.1426(9929)

S = 1.008 Npar= 595

The following ALERTS were generated. Each ALERT has the format
test-name_ALERT_alert-type_alert-level.
Click on the hyperlinks for more details of the test.

Alert level B

PLAT043_ALERT_1_B Calculated and Reported Mol. Weight Differ by .. 12.08 Check

Author Response: Due to the caculation of disordered H atoms on solvent water molecules

PLAT306_ALERT_2_B Isolated Oxygen Atom (H-atoms Missing ?) .... O1W Check

Author Response: Due to the disorder of H atoms on solvent water molecules.

PLAT306_ALERT_2_B Isolated Oxygen Atom (H-atoms Missing ?) .... O2W Check

Author Response: Due to the disorder of H atoms on solvent water molecules.

PLAT306_ALERT_2_B Isolated Oxygen Atom (H-atoms Missing ?) .... O3W Check

Author Response: Due to the disorder of H atoms on solvent water molecules.

PLAT306_ALERT_2_B Isolated Oxygen Atom (H-atoms Missing ?) .... O4W Check

Author Response: Due to the disorder of H atoms on solvent water molecules.

PLAT306_ALERT_2_B Isolated Oxygen Atom (H-atoms Missing ?) .... O5W Check

Author Response: Due to the disorder of H atoms on solvent water molecules.

PLAT306_ALERT_2_B Isolated Oxygen Atom (H-atoms Missing ?) .... O6W Check

Author Response: Due to the disorder of H atoms on solvent water molecules.

PLAT430_ALERT_2_B Short Inter D...A Contact O1W ..O11 . 2.72 Ang.
x,y,z = 1_555 Check

Author Response: Due to the intermolecular hydregen bonding interaction.

PLAT430_ALERT_2_B Short Inter D...A Contact O1W ..O2W . 2.80 Ang.
x,y,z = 1_555 Check

Author Response: Due to the intermolecular hydregen bonding interaction.

PLAT430_ALERT_2_B Short Inter D...A Contact O1W ..O4W . 2.83 Ang.
x,y,z = 1_555 Check

Author Response: Due to the intermolecular hydrogen bonding interaction.

```
PLAT430_ALERT_2_B Short Inter D...A Contact  O2W      ..O8       .       2.82 Ang.
                                                -1+x,y,z  =      1_455 Check
```

Author Response: Due to the intermolecular hydrogen bonding interaction.

```
PLAT430_ALERT_2_B Short Inter D...A Contact  O3W      ..O6W      .       2.84 Ang.
                                              1-x,1-y,-z  =      2_665 Check
```

Author Response: Due to the intermolecular hydrogen bonding interaction.

```
PLAT430_ALERT_2_B Short Inter D...A Contact  O4W      ..O5W      .       2.78 Ang.
                                                   x,y,z  =      1_555 Check
```

Author Response: Due to the intermolecular hydrogen bonding interaction.

```
PLAT430_ALERT_2_B Short Inter D...A Contact  O5W      ..O5W      .       2.68 Ang.
                                              1-x,1-y,-z  =      2_665 Check
```

Author Response: Due to the intermolecular hydrogen bonding interaction.

```
PLAT430_ALERT_2_B Short Inter D...A Contact  O5W      ..O6W      .       2.76 Ang.
                                                   x,y,z  =      1_555 Check
```

Author Response: Due to the intermolecular hydrogen bonding interaction.

```
PLAT430_ALERT_2_B Short Inter D...A Contact  O6W      ..O7       .       2.77 Ang.
                                                   x,y,z  =      1_555 Check
```

Author Response: Due to the intermolecular hydrogen bonding interaction.

Alert level C

```
PLAT041_ALERT_1_C Calc. and Reported SumFormula    Strings  Differ     Please Check
PLAT044_ALERT_1_C Calculated and Reported Density Dx  Differ by ..     0.0186 Check
PLAT048_ALERT_1_C MoietyFormula Not Given (or Incomplete) ........     Please Check
PLAT057_ALERT_3_C Correction for Absorption Required   RT(exp) ...       1.18 Do !
PLAT068_ALERT_1_C Reported F000 Differs from Calcd (or Missing)...     Please Check
PLAT241_ALERT_2_C High   'MainMol' Ueq as Compared to Neighbors of         O2 Check
PLAT260_ALERT_2_C Large Average Ueq of Residue Including      O6W      0.106 Check
PLAT369_ALERT_2_C Long   C(sp2)-C(sp2) Bond  C20      - C22      .       1.53 Ang.
PLAT430_ALERT_2_C Short Inter D...A Contact  O3W      ..O4W      .       2.85 Ang.
                                                   x,y,z  =      1_555 Check
```

Author Response: Due to the intermolecular hydrogen bonding interaction.

```
PLAT911_ALERT_3_C Missing FCF Refl Between Thmin & STh/L=    0.600          3 Report
PLAT971_ALERT_2_C Check Calcd Resid. Dens.  0.21A   From O6W             1.66 eA-3
PLAT972_ALERT_2_C Check Calcd Resid. Dens.  0.74A   From Pb1            -1.78 eA-3
PLAT972_ALERT_2_C Check Calcd Resid. Dens.  0.81A   From Pb1            -1.61 eA-3
PLAT975_ALERT_2_C Check Calcd Resid. Dens.  0.78A   From O2W             0.86 eA-3
PLAT975_ALERT_2_C Check Calcd Resid. Dens.  1.00A   From O1W             0.63 eA-3
PLAT975_ALERT_2_C Check Calcd Resid. Dens.  0.52A   From O5              0.59 eA-3
PLAT976_ALERT_2_C Check Calcd Resid. Dens.  0.46A   From O6W            -1.40 eA-3
PLAT976_ALERT_2_C Check Calcd Resid. Dens.  0.85A   From O6W            -0.87 eA-3
PLAT976_ALERT_2_C Check Calcd Resid. Dens.  0.53A   From O4W            -0.74 eA-3
PLAT976_ALERT_2_C Check Calcd Resid. Dens.  0.53A   From O5W            -0.59 eA-3
PLAT976_ALERT_2_C Check Calcd Resid. Dens.  0.68A   From O5W            -0.59 eA-3
PLAT976_ALERT_2_C Check Calcd Resid. Dens.  0.60A   From O4W            -0.57 eA-3
PLAT976_ALERT_2_C Check Calcd Resid. Dens.  0.61A   From O10            -0.57 eA-3
PLAT977_ALERT_2_C Check Negative Difference Density on H41A             -0.46 eA-3
PLAT977_ALERT_2_C Check Negative Difference Density on H41B             -0.49 eA-3
PLAT977_ALERT_2_C Check Negative Difference Density on H41C             -0.34 eA-3
PLAT977_ALERT_2_C Check Negative Difference Density on H42B             -0.43 eA-3
PLAT977_ALERT_2_C Check Negative Difference Density on H42C             -0.51 eA-3
```

Alert level G

```
FORMU01_ALERT_2_G  There is a discrepancy between the atom counts in the
            _chemical_formula_sum and the formula from the _atom_site* data.
            Atom count from _chemical_formula_sum:C84 H94 N12 O34 Pb2
            Atom count from the _atom_site data:  C84 H70 N12 O34 Pb2
CELLZ01_ALERT_1_G Difference between formula and atom_site contents detected.
CELLZ01_ALERT_1_G WARNING: H atoms missing from atom site list. Is this intentional?
           From the CIF: _cell_formula_units_Z    1
           From the CIF: _chemical_formula_sum  C84 H94 N12 O34 Pb2
           TEST: Compare cell contents of formula and atom_site data

           atom    Z*formula  cif sites diff
           C         84.00     84.00    0.00
           H         94.00     70.00   24.00
           N         12.00     12.00    0.00
           O         34.00     34.00    0.00
           Pb         2.00      2.00    0.00
PLAT004_ALERT_5_G Polymeric Structure Found with Maximum Dimension          2 Info
PLAT007_ALERT_5_G Number of Unrefined Donor-H Atoms .............           1 Report
PLAT045_ALERT_1_G Calculated and Reported Z Differ by a Factor ...       2.00 Check
PLAT154_ALERT_1_G The s.u.’s on the Cell Angles are Equal ..(Note)       0.03 Degree
PLAT380_ALERT_4_G Incorrectly? Oriented X(sp2)-Methyl Moiety .....        C41 Check
PLAT380_ALERT_4_G Incorrectly? Oriented X(sp2)-Methyl Moiety .....        C42 Check
PLAT432_ALERT_2_G Short Inter X...Y Contact  C6       ..C36              3.10 Ang.
                                                    x,y,z  =      1_555 Check
PLAT794_ALERT_5_G Tentative Bond Valency for Pb1       (II)      .       2.08 Info
PLAT802_ALERT_4_G CIF Input Record(s) with more than 80 Characters          1 Info
PLAT883_ALERT_1_G No Info/Value for _atom_sites_solution_primary .     Please Do !
PLAT910_ALERT_3_G Missing # of FCF Reflection(s) Below Theta(Min).          2 Note
PLAT912_ALERT_4_G Missing # of FCF Reflections Above STh/L=  0.600         10 Note
PLAT913_ALERT_3_G Missing # of Very Strong Reflections in FCF ....          1 Note
PLAT941_ALERT_3_G Average HKL Measurement Multiplicity ...........        3.2 Low
PLAT965_ALERT_2_G The SHELXL WEIGHT Optimisation has not Converged     Please Check
PLAT978_ALERT_2_G Number C-C Bonds with Positive Residual Density.          0 Info
```

---

0 **ALERT level A** = Most likely a serious problem - resolve or explain
16 **ALERT level B** = A potentially serious problem, consider carefully
28 **ALERT level C** = Check. Ensure it is not caused by an omission or oversight
19 **ALERT level G** = General information/check it is not something unexpected

```
10 ALERT type 1 CIF construction/syntax error, inconsistent or missing data
41 ALERT type 2 Indicator that the structure model may be wrong or deficient
 5 ALERT type 3 Indicator that the structure quality may be low
 4 ALERT type 4 Improvement, methodology, query or suggestion
 3 ALERT type 5 Informative message, check
```

Publication of your CIF

You should attempt to resolve as many as possible of the alerts in all categories. Often the minor alerts point to easily fixed oversights, errors and omissions in your CIF or refinement strategy, so attention to these fine details can be worthwhile. In order to resolve some of the more serious problems it may be necessary to carry out additional measurements or structure refinements. However, the nature of your study may justify the reported deviations from journal submission requirements and the more serious of these should be commented upon in the discussion or experimental section of a paper or in the "special_details" fields of the CIF. *checkCIF* was carefully designed to identify outliers and unusual parameters, but every test has its limitations and alerts that are not important in a particular case may appear. Conversely, the absence of alerts does not guarantee there are no aspects of the results needing attention. It is up to the individual to critically assess their own results and, if necessary, seek expert advice.

If you wish to submit your CIF for publication in Acta Crystallographica Section C or E, you should upload your CIF via the web. If you wish to submit your CIF for publication in IUCrData you should upload your CIF via the web. If your CIF is to form part of a submission to another IUCr journal, you will be asked, either during electronic submission or by the Co-editor handling your paper, to upload your CIF via our web site.

PLATON version of 03/06/2021; check.def file version of 02/06/2021

Datablock I - ellipsoid plot

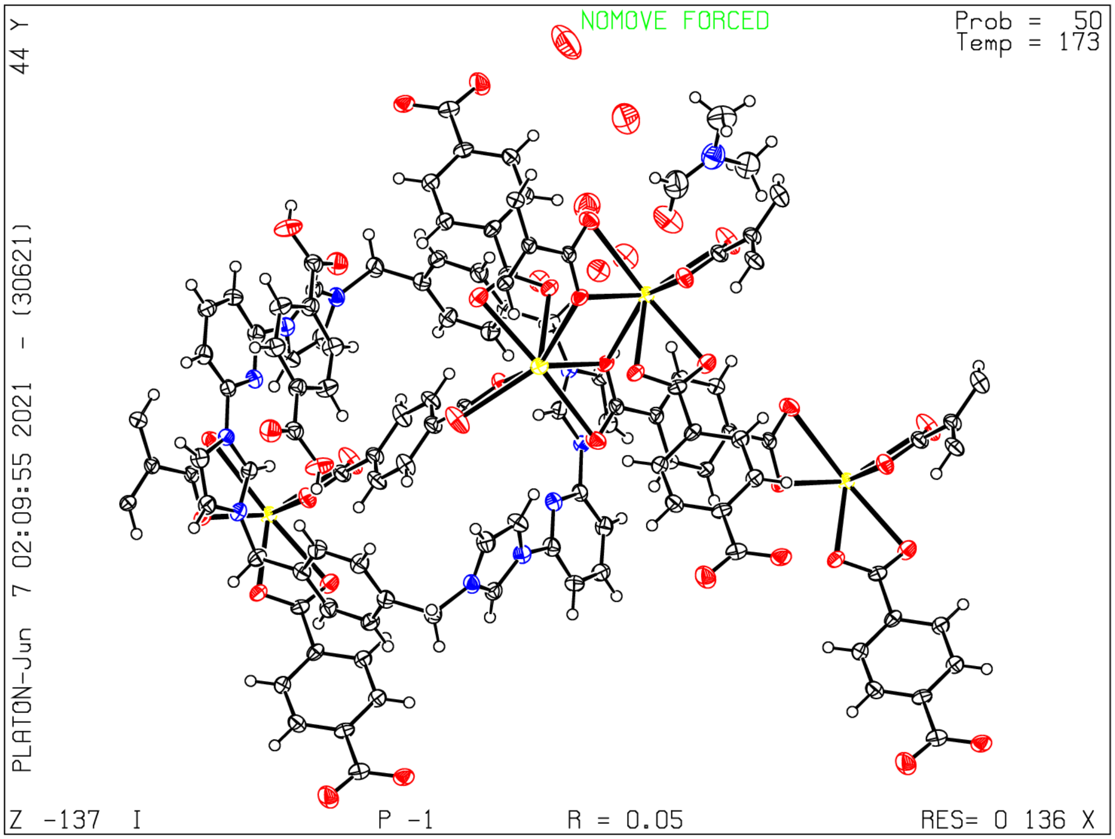

Supplement: Supplementary file 1 [file molecules-26-04241-s001.zip › MORF-Pb-1-checkcif.pdf]
