# Supplementary material for: Pb2+-Containing Metal-Organic Rotaxane Frameworks (MORFs)
Source: Molecules. 2021 Jul 13;26(14):4241. doi: 10.3390/molecules26144241 (PMC8306753; doi:10.3390/molecules26144241)

checkCIF/PLATON report

Structure factors have been supplied for datablock(s) I

THIS REPORT IS FOR GUIDANCE ONLY. IF USED AS PART OF A REVIEW PROCEDURE FOR PUBLICATION, IT SHOULD NOT REPLACE THE EXPERTISE OF AN EXPERIENCED CRYSTALLOGRAPHIC REFEREE.

No syntax errors found.   CIF dictionary   Interpreting this report

Datablock: I

```
Bond precision:  C-C = 0.0251 A          Wavelength=0.71073

Cell:            a=20.504(4)     b=20.005(4)        c=24.046(5)
                 alpha=90        beta=101.23(3)     gamma=90
Temperature:     153 K

                Calculated                 Reported
Volume          9674(4)                    9674(3)
Space group     P 21/n                     P 21/n
Hall group      -P 2yn                     -P 2yn
                2(C60 H30 O21 Pb3), 2(C38
Moiety formula  H34 N10), 20(O0.50),       ?
                2(O0.50), 4(O0.5
Sum formula     C196 H128 N20 O67 Pb6      C98 H89 N10 O32.50 Pb3
Mr              5078.39                    2548.36
Dx,g cm-3       1.743                      1.750
Z               2                          4
Mu (mm-1)       5.299                      5.298
F000            4944.0                     5012.0
F000’           4906.53
h,k,lmax        24,23,28                   24,23,28
Nref            17037                      16968
Tmin,Tmax       0.471,0.589                0.733,1.000
Tmin’           0.325

Correction method= # Reported T Limits: Tmin=0.733 Tmax=1.000
AbsCorr = MULTI-SCAN

Data completeness= 0.996          Theta(max)= 24.999

R(reflections)= 0.0789( 9354)     wR2(reflections)= 0.2551( 16968)

S = 1.006                    Npar= 1114
```

The following ALERTS were generated. Each ALERT has the format
test-name_ALERT_alert-type_alert-level.
Click on the hyperlinks for more details of the test.

Alert level A

```
PLAT430_ALERT_2_A Short Inter D...A Contact  O11W     ..O11W     .       1.83 Ang.
                                                 1-x,-y,1-z  =      3_656 Check
```

Author Response: Due to the distribution disorder of the solvent molecule or possible i

```
PLAT430_ALERT_2_A Short Inter D...A Contact  O14W     ..O15W     .       2.46 Ang.
                                         1/2-x,-1/2+y,1/2-z  =      2_545 Check
```

Author Response: Due to the distribution disorder of the solvent molecule or possible i

```
PLAT973_ALERT_2_A Check Calcd Positive Resid. Density on       Pb1       2.79 eA-3
```

Author Response: Minor residual density near Pb is not unusual.

```
PLAT973_ALERT_2_A Check Calcd Positive Resid. Density on       Pb2       2.36 eA-3
```

Author Response: Minor residual density near Pb is not unusual.

```
PLAT973_ALERT_2_A Check Calcd Positive Resid. Density on       Pb3       2.19 eA-3
```

Author Response: Minor residual density near Pb is not unusual.

```
PLAT973_ALERT_2_A Check Calcd Positive Resid. Density on       Pb3       2.19 eA-3
```

Author Response: Minor residual density near Pb is not unusual.

Alert level B

```
PLAT306_ALERT_2_B Isolated Oxygen Atom (H-atoms Missing ?) .......        O1W Check
```

Author Response: Due to the disorder of H atoms on solvent water molecules.

```
PLAT306_ALERT_2_B Isolated Oxygen Atom (H-atoms Missing ?) .......        O2W Check
```

Author Response: Due to the disorder of H atoms on solvent water molecules.

```
PLAT306_ALERT_2_B Isolated Oxygen Atom (H-atoms Missing ?) .......        O3W Check
```

Author Response: Due to the disorder of H atoms on solvent water molecules.

```
PLAT306_ALERT_2_B Isolated Oxygen Atom (H-atoms Missing ?) .......        O4W Check
```

Author Response: Due to the disorder of H atoms on solvent water molecules.

```
PLAT306_ALERT_2_B Isolated Oxygen Atom (H-atoms Missing ?) .......        O5W Check
```

Author Response: Due to the disorder of H atoms on solvent water molecules.

```
PLAT306_ALERT_2_B Isolated Oxygen Atom (H-atoms Missing ?) .......        O6W Check
```

Author Response: Due to the disorder of H atoms on solvent water molecules.

```
PLAT306_ALERT_2_B Isolated Oxygen Atom (H-atoms Missing ?) .......        O8W Check
```

Author Response: Due to the disorder of H atoms on solvent water molecules.

```
PLAT306_ALERT_2_B Isolated Oxygen Atom (H-atoms Missing ?) .......        O9W Check
```

Author Response: Due to the disorder of H atoms on solvent water molecules.

```
PLAT306_ALERT_2_B Isolated Oxygen Atom (H-atoms Missing ?) .......       O10W Check
```

Author Response: Due to the disorder of H atoms on solvent water molecules.

```
PLAT306_ALERT_2_B Isolated Oxygen Atom (H-atoms Missing ?) .......       O13W Check
```

Author Response: Due to the disorder of H atoms on solvent water molecules.

```
PLAT342_ALERT_3_B Low Bond Precision on  C-C Bonds ...............    0.02505 Ang.
```

Author Response: Due to the molecular disorder.

```
PLAT369_ALERT_2_B Long   C(sp2)-C(sp2) Bond  C81      - C86      .       1.58 Ang.
```

Author Response: It is a normal C-C single bond.

```
PLAT430_ALERT_2_B Short Inter D...A Contact  O1W      ..O13      .       2.79 Ang.
                                                      x,y,z  =      1_555 Check
```

Author Response: Due to the distribution disorder of the solvent molecule or possible i

```
PLAT430_ALERT_2_B Short Inter D...A Contact  O2       ..O15W     .       2.61 Ang.
                                                      x,y,z  =      1_555 Check
```

Author Response: Due to the distribution disorder of the solvent molecule or possible i

```
PLAT430_ALERT_2_B Short Inter D...A Contact  O2W      ..O8W      .       2.71 Ang.
                                                     x,y,z  =      1_555 Check
```

Author Response: Due to the distribution disorder of the solvent molecule or possible i

```
PLAT430_ALERT_2_B Short Inter D...A Contact  O3W      ..O14      .       2.78 Ang.
                                                     x,y,z  =      1_555 Check
```

Author Response: Due to the distribution disorder of the solvent molecule or possible i

```
PLAT430_ALERT_2_B Short Inter D...A Contact  O4       ..O16W     .       2.69 Ang.
                                                     x,y,z  =      1_555 Check
```

Author Response: Due to the distribution disorder of the solvent molecule or possible i

```
PLAT430_ALERT_2_B Short Inter D...A Contact  O5       ..O5W      .       2.72 Ang.
                                         1/2+x,1/2-y,1/2+z  =      4_666 Check
```

Author Response: Due to the distribution disorder of the solvent molecule or possible i

```
PLAT430_ALERT_2_B Short Inter D...A Contact  O5W      ..O6W      .       2.77 Ang.
                                                 -x,-y,1-z  =      3_556 Check
```

Author Response: Due to the distribution disorder of the solvent molecule or possible i

```
PLAT430_ALERT_2_B Short Inter D...A Contact  O5W      ..O5W      .       2.80 Ang.
                                                 -x,-y,1-z  =      3_556 Check
```

Author Response: Due to the distribution disorder of the solvent molecule or possible i

```
PLAT430_ALERT_2_B Short Inter D...A Contact  O7       ..O10W     .       2.68 Ang.
                                               1-x,1-y,1-z  =      3_666 Check
```

Author Response: Due to the distribution disorder of the solvent molecule or possible i

```
PLAT430_ALERT_2_B Short Inter D...A Contact  O7W      ..O15W     .       2.75 Ang.
                                       -1/2+x,3/2-y,-1/2+z  =      4_575 Check
```

Author Response: Due to the distribution disorder of the solvent molecule or possible i

```
PLAT430_ALERT_2_B Short Inter D...A Contact  O8W      ..O18      .       2.80 Ang.
                                                     x,y,z  =      1_555 Check
```

Author Response: Due to the distribution disorder of the solvent molecule or possible i

```
PLAT430_ALERT_2_B Short Inter D...A Contact  O9W      ..O13W     .       2.79 Ang.
                                        1/2-x,-1/2+y,3/2-z  =      2_546 Check
```

Author Response: Due to the distribution disorder of the solvent molecule or possible i

```
PLAT430_ALERT_2_B Short Inter D...A Contact  O10      ..O11W     .       2.77 Ang.
                                               1-x,-y,1-z  =      3_656 Check
```

Author Response: Due to the distribution disorder of the solvent molecule or possible i

```
PLAT430_ALERT_2_B Short Inter D...A Contact  O10W     ..O10W     .       2.72 Ang.
                                              1-x,1-y,1-z  =      3_666 Check
```

Author Response: Due to the distribution disorder of the solvent molecule or possible i

```
PLAT430_ALERT_2_B Short Inter D...A Contact  O10W     ..O13W     .       2.79 Ang.
                                                    x,y,z  =      1_555 Check
```

Author Response: Due to the distribution disorder of the solvent molecule or possible i

```
PLAT430_ALERT_2_B Short Inter D...A Contact  O11W     ..O12W     .       2.59 Ang.
                                                    x,y,z  =      1_555 Check
```

Author Response: Due to the distribution disorder of the solvent molecule or possible i

```
PLAT430_ALERT_2_B Short Inter D...A Contact  O11W     ..O15W     .       2.73 Ang.
                                              1-x,1-y,1-z  =      3_666 Check
```

Author Response: Due to the distribution disorder of the solvent molecule or possible i

```
PLAT430_ALERT_2_B Short Inter D...A Contact  O13W     ..O16W     .       2.60 Ang.
                                      -1/2+x,3/2-y,-1/2+z  =      4_575 Check
```

Author Response: Due to the distribution disorder of the solvent molecule or possible i

```
PLAT430_ALERT_2_B Short Inter D...A Contact  O13W     ..O17      .       2.84 Ang.
                                               -x,1-y,1-z  =      3_566 Check
```

Author Response: Due to the distribution disorder of the solvent molecule or possible i

```
PLAT780_ALERT_1_B Coordinates do not Form a Properly Connected Set     Please Do !
```

Author Response: Due to the complex coordination of Pb cation.

```
PLAT972_ALERT_2_B Check Calcd Resid. Dens.  0.93A   From Pb3            -3.13 eA-3
```

Author Response: Minor residual density near Pb is not unusual.

Alert level C

```
ABSTY02_ALERT_1_C  An _exptl_absorpt_correction_type has been given without
            a literature citation. This should be contained in the
            _exptl_absorpt_process_details field.
            Absorption correction given as Multi-scan
RINTA01_ALERT_3_C  The value of Rint is greater than 0.12
            Rint given   0.152
PLAT018_ALERT_1_C _diffrn_measured_fraction_theta_max .NE. *_full          ! Check
PLAT020_ALERT_3_C The Value of Rint is Greater Than 0.12 .........      0.152 Report
PLAT041_ALERT_1_C Calc. and Reported SumFormula    Strings  Differ     Please Check
PLAT043_ALERT_1_C Calculated and Reported Mol. Weight Differ by ..      18.33 Check
PLAT048_ALERT_1_C MoietyFormula Not Given (or Incomplete) ........     Please Check
PLAT068_ALERT_1_C Reported F000 Differs from Calcd (or Missing)...     Please Check
PLAT084_ALERT_3_C High wR2 Value (i.e. > 0.25) ...................       0.26 Report
PLAT241_ALERT_2_C High   'MainMol' Ueq as Compared to Neighbors of        O5 Check
PLAT241_ALERT_2_C High   'MainMol' Ueq as Compared to Neighbors of       O10 Check
PLAT241_ALERT_2_C High   'MainMol' Ueq as Compared to Neighbors of       O14 Check
PLAT241_ALERT_2_C High   'MainMol' Ueq as Compared to Neighbors of       O15 Check
PLAT241_ALERT_2_C High   'MainMol' Ueq as Compared to Neighbors of       C33 Check
PLAT242_ALERT_2_C Low    'MainMol' Ueq as Compared to Neighbors of       Pb1 Check
PLAT242_ALERT_2_C Low    'MainMol' Ueq as Compared to Neighbors of       O16 Check
PLAT242_ALERT_2_C Low    'MainMol' Ueq as Compared to Neighbors of       C39 Check
PLAT242_ALERT_2_C Low    'MainMol' Ueq as Compared to Neighbors of       C98 Check
PLAT242_ALERT_2_C Low    'MainMol' Ueq as Compared to Neighbors of       C13 Check
PLAT242_ALERT_2_C Low    'MainMol' Ueq as Compared to Neighbors of       C32 Check
PLAT260_ALERT_2_C Large Average Ueq of Residue Including      O8W      0.118 Check
PLAT260_ALERT_2_C Large Average Ueq of Residue Including      O9W      0.133 Check
PLAT260_ALERT_2_C Large Average Ueq of Residue Including     O11W      0.101 Check
PLAT260_ALERT_2_C Large Average Ueq of Residue Including     O13W      0.137 Check
PLAT260_ALERT_2_C Large Average Ueq of Residue Including     O14W      0.135 Check
PLAT260_ALERT_2_C Large Average Ueq of Residue Including     O15W      0.130 Check
PLAT260_ALERT_2_C Large Average Ueq of Residue Including     O16W      0.141 Check
PLAT334_ALERT_2_C Small Aver. Benzene C-C Dist C13      -C18            1.37 Ang.
PLAT334_ALERT_2_C Small Aver. Benzene C-C Dist C32      -C37            1.37 Ang.
PLAT430_ALERT_2_C Short Inter D...A Contact  O1W      ..O2W      .      2.87 Ang.
                                          1/2+x,1/2-y,1/2+z  =      4_666 Check
```

Author Response: Due to the distribution disorder of the solvent molecule or possible i

```
PLAT430_ALERT_2_C Short Inter D...A Contact  O4W      ..O9W      .      2.85 Ang.
                                         1/2-x,-1/2+y,3/2-z  =      2_546 Check
```

Author Response: Due to the distribution disorder of the solvent molecule or possible i

```
PLAT430_ALERT_2_C Short Inter D...A Contact  O6W      ..O16      .      2.85 Ang.
                                                     x,y,z  =      1_555 Check
```

Author Response: Due to the distribution disorder of the solvent molecule or possible i

```
PLAT430_ALERT_2_C Short Inter D...A Contact  O9W      ..O19      .      2.87 Ang.
                                             -x,1-y,1-z  =      3_566 Check
```

Author Response: Due to the distribution disorder of the solvent molecule or possible i

PLAT906_ALERT_3_C Large K Value in the Analysis of Variance ...... 5.474 Check
PLAT911_ALERT_3_C Missing FCF Refl Between Thmin & STh/L= 0.595 69 Report
PLAT971_ALERT_2_C Check Calcd Resid. Dens. 2.74A From O2 2.12 eA-3
PLAT971_ALERT_2_C Check Calcd Resid. Dens. 0.53A From Pb3 1.74 eA-3
PLAT972_ALERT_2_C Check Calcd Resid. Dens. 0.81A From Pb2 -1.54 eA-3

Author Response: Minor residual density near Pb is not unusual.

PLAT976_ALERT_2_C Check Calcd Resid. Dens. 0.76A From O11 -0.94 eA-3
PLAT977_ALERT_2_C Check Negative Difference Density on H6A -0.58 eA-3
PLAT977_ALERT_2_C Check Negative Difference Density on H41A -0.36 eA-3

Alert level G

FORMU01_ALERT_2_G There is a discrepancy between the atom counts in the
_chemical_formula_sum and the formula from the _atom_site* data.
Atom count from _chemical_formula_sum:C98 H89 N10 O32.5 Pb3
Atom count from the _atom_site data: C98 H64 N10 O33.5 Pb3
CELLZ01_ALERT_1_G Difference between formula and atom_site contents detected.
CELLZ01_ALERT_1_G ALERT: Large difference may be due to a
symmetry error - see SYMMG tests
From the CIF: _cell_formula_units_Z 4
From the CIF: _chemical_formula_sum C98 H89 N10 O32.50 Pb3
TEST: Compare cell contents of formula and atom_site data

atom	Z*formula	cif sites	diff
C	392.00	392.00	0.00
H	356.00	256.00	100.00
N	40.00	40.00	0.00
O	130.00	134.00	-4.00
Pb	12.00	12.00	0.00

PLAT002_ALERT_2_G Number of Distance or Angle Restraints on AtSite 12 Note
PLAT003_ALERT_2_G Number of Uiso or Uij Restrained non-H Atoms ... 15 Report
PLAT004_ALERT_5_G Polymeric Structure Found with Maximum Dimension 3 Info
PLAT045_ALERT_1_G Calculated and Reported Z Differ by a Factor ... 0.50 Check
PLAT083_ALERT_2_G SHELXL Second Parameter in WGHT Unusually Large 20.00 Why ?
PLAT171_ALERT_4_G The CIF-Embedded .res File Contains EADP Records 6 Report
PLAT172_ALERT_4_G The CIF-Embedded .res File Contains DFIX Records 8 Report
PLAT186_ALERT_4_G The CIF-Embedded .res File Contains ISOR Records 1 Report
PLAT300_ALERT_4_G Atom Site Occupancy of O11W Constrained at 0.5 Check
PLAT300_ALERT_4_G Atom Site Occupancy of O12W Constrained at 0.5 Check
PLAT300_ALERT_4_G Atom Site Occupancy of O14W Constrained at 0.5 Check
PLAT300_ALERT_4_G Atom Site Occupancy of O15W Constrained at 0.5 Check
PLAT300_ALERT_4_G Atom Site Occupancy of O16W Constrained at 0.5 Check
PLAT302_ALERT_4_G Anion/Solvent/Minor-Residue Disorder (Resd 12) 100% Note
PLAT302_ALERT_4_G Anion/Solvent/Minor-Residue Disorder (Resd 13) 100% Note
PLAT302_ALERT_4_G Anion/Solvent/Minor-Residue Disorder (Resd 15) 100% Note
PLAT302_ALERT_4_G Anion/Solvent/Minor-Residue Disorder (Resd 16) 100% Note
PLAT302_ALERT_4_G Anion/Solvent/Minor-Residue Disorder (Resd 17) 100% Note
PLAT311_ALERT_2_G Isolated Disordered Oxygen Atom (No H’s ?) ..... O11W Check
PLAT311_ALERT_2_G Isolated Disordered Oxygen Atom (No H’s ?) ..... O12W Check
PLAT311_ALERT_2_G Isolated Disordered Oxygen Atom (No H’s ?) ..... O14W Check
PLAT311_ALERT_2_G Isolated Disordered Oxygen Atom (No H’s ?) ..... O15W Check
PLAT311_ALERT_2_G Isolated Disordered Oxygen Atom (No H’s ?) ..... O16W Check
PLAT335_ALERT_2_G Check Large C6 Ring C-C Range C76 -C85 0.26 Ang.
PLAT335_ALERT_2_G Check Large C6 Ring C-C Range C79 -C84 0.19 Ang.
PLAT432_ALERT_2_G Short Inter X...Y Contact O12 ..C9 2.97 Ang.
1/2+x,1/2-y,1/2+z = 4_666 Check
PLAT432_ALERT_2_G Short Inter X...Y Contact O17 ..C9 2.96 Ang.
-x,1-y,1-z = 3_566 Check

```
PLAT432_ALERT_2_G Short Inter X...Y Contact  C25      ..C60            3.20 Ang.
                                        -1/2+x,1/2-y,-1/2+z  =      4_565 Check
PLAT794_ALERT_5_G Tentative Bond Valency for Pb1       (II)      .       2.00 Info
PLAT794_ALERT_5_G Tentative Bond Valency for Pb3       (II)      .       1.91 Info
PLAT802_ALERT_4_G CIF Input Record(s) with more than 80 Characters          1 Info
PLAT860_ALERT_3_G Number of Least-Squares Restraints ............         123 Note
PLAT883_ALERT_1_G No Info/Value for _atom_sites_solution_primary .     Please Do !
PLAT910_ALERT_3_G Missing # of FCF Reflection(s) Below Theta(Min).          2 Note
PLAT933_ALERT_2_G Number of OMIT Records in Embedded .res File ...         30 Note
PLAT941_ALERT_3_G Average HKL Measurement Multiplicity ...........        3.9 Low
PLAT960_ALERT_3_G Number of Intensities with I  <   - 2*sig(I) ...         40 Check
PLAT965_ALERT_2_G The SHELXL WEIGHT Optimisation has not Converged     Please Check
PLAT978_ALERT_2_G Number C-C Bonds with Positive Residual Density.          1 Info
```

```
  6 ALERT level A = Most likely a serious problem - resolve or explain
 33 ALERT level B = A potentially serious problem, consider carefully
 41 ALERT level C = Check. Ensure it is not caused by an omission or oversight
 42 ALERT level G = General information/check it is not something unexpected

 11 ALERT type 1 CIF construction/syntax error, inconsistent or missing data
 84 ALERT type 2 Indicator that the structure model may be wrong or deficient
 10 ALERT type 3 Indicator that the structure quality may be low
 14 ALERT type 4 Improvement, methodology, query or suggestion
  3 ALERT type 5 Informative message, check
```

Publication of your CIF

You should attempt to resolve as many as possible of the alerts in all categories. Often the minor alerts point to easily fixed oversights, errors and omissions in your CIF or refinement strategy, so attention to these fine details can be worthwhile. In order to resolve some of the more serious problems it may be necessary to carry out additional measurements or structure refinements. However, the nature of your study may justify the reported deviations from journal submission requirements and the more serious of these should be commented upon in the discussion or experimental section of a paper or in the "special_details" fields of the CIF. *checkCIF* was carefully designed to identify outliers and unusual parameters, but every test has its limitations and alerts that are not important in a particular case may appear. Conversely, the absence of alerts does not guarantee there are no aspects of the results needing attention. It is up to the individual to critically assess their own results and, if necessary, seek expert advice.

If you wish to submit your CIF for publication in Acta Crystallographica Section C or E, you should upload your CIF via the web. If you wish to submit your CIF for publication in IUCrData, you should upload your CIF via the web. If your CIF is to form part of a submission to another IUCr journal, you will be asked, either during electronic submission or by the Co-editor handling your paper, to upload your CIF via our web site.

PLATON version of 22/03/2021; check.def file version of 19/03/2021

Datablock I - ellipsoid plot

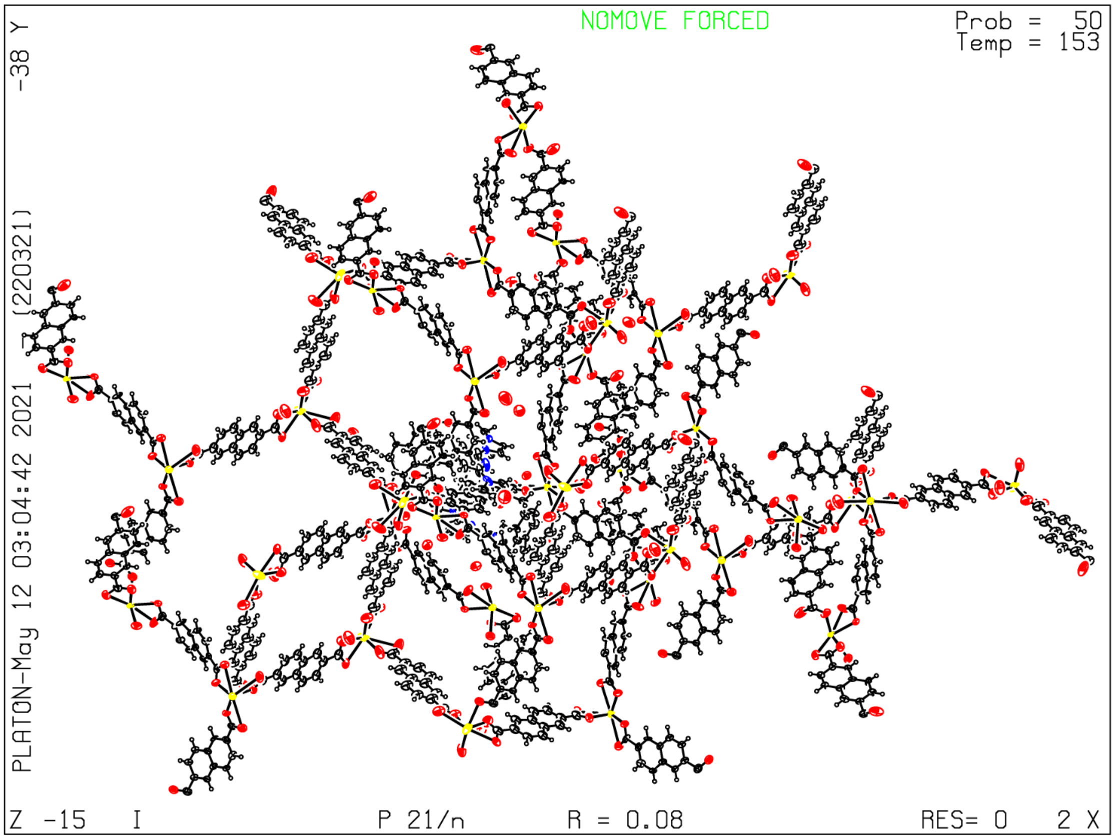

Supplement: Supplementary file 1 [file molecules-26-04241-s001.zip › MORF-Pb-2-checkcif.pdf]
